# 肺良性转移性平滑肌瘤5例报道

**DOI:** 10.3779/j.issn.1009-3419.2014.07.09

**Published:** 2014-07-20

**Authors:** 键 冯, 波 叶, 煜 杨, 旭峰 潘, 凌 林, 勇 陈, 克坚 曹, 建新 施, 珩 赵

**Affiliations:** 200030 上海，上海市胸科医院，上海交通大学附属胸科医院胸外科 Department of Thoracic Surgery, Shanghai Chest Hospital, Shanghai Jiao Tong University, Shanghai 200030, China

**Keywords:** 良性转移性平滑肌瘤, 肺肿瘤, 临床病理, 转移, Benign metastasizing leiomyoma, Lung neoplasms, Clinical pathology, Metastasis

## Abstract

肺良性转移性平滑肌瘤（pulmonary benign metastasizing leiomyoma, PBML）是一种罕见疾病，临床工作中易被误诊。本文拟探讨PBML的临床病理学特征、诊断、治疗及预后。回顾性分析5例PBML患者的临床资料和病理学特点。患者均为女性，平均年龄46.8岁。肺转移瘤发生在诊断子宫平滑肌瘤后89个月-226个月。4例为肺内多发性结节，1例为单发性，1例伴左肾转移。手术方式包括3例胸腔镜下楔形切除，1例左肺上叶切除，1例右肺上叶切除+右肺中叶、下叶楔形切除。术后随访3个月-48个月，肺内残余病灶无进展。PBML主要发生于有子宫平滑肌瘤病史的女性。肺是最主要的转移部位。手术切除是主要的治疗手段。肿瘤激素依赖性，可予内分泌治疗。

## Abstract (en)

Pulmonary benign metastasizing leiomyoma (PBML) is a rare and often to be misdiagnosed disease. In this study, we want to investigate the diseased of the clinicopathological characteristics, diagnosis, treatment and prognosis. The retrospective analysis were performed on the clinicopathological data of 5 patients with PBML. All 5 cases were female, mean age 46.8 years old, and were found with single nodule. One case was found with left kidney metastasis. Surgical procedures includes: VATS biopsy (3 cases), resection of superior lobe of left lung (1 case), resection of superior lobe of right lung and wedge resection of middle and inferior lobe of right lung (1 case). The residual nodules in lung were stable in all cases with followup 3-48 mo. PBML is dominated occurring in females with history of uterine leiomyoma, preferential to metastasize to lung, and surgery is the primary therapy. Since it is hormone dependent, hormonal therapy may be suggested in these patients.

肺良性转移性平滑肌瘤（pulmonary benign metastasizing leiomyoma, PBML）是一种临床工作中十分罕见的肺内转移性良性肿瘤，迄今为止只有少数报道，其在影像学表现上往往为肺内多发性病灶，因此临床工作中较易被诊断为肺恶性肿瘤导致的肺内播散。现报道本院收治的5例PBML患者，以探讨本病的临床、病理特点及诊治要点。

## 1 临床资料

2008年4月-2014年1月上海市胸科医院共收治肺良性转移性平滑肌瘤病患者5例，收集患者临床病理资料和免疫组化结果进行分析，并于2014年1月通过电话集中进行随访。

5例患者均为女性，年龄43岁-52岁，平均46.8岁，均无明显临床症状，体检发现肺部病变。患者均有子宫平滑肌瘤病史，其中4例有子宫肌瘤手术史，1例未行手术治疗。肺良性转移性平滑肌瘤发生在诊断子宫平滑肌瘤后89个月-226个月，平均169个月。胸部计算机断层扫描（computed tomography, CT）检查提示4例患者为肺内多发性结节，其中1例患者包含有偏心性空洞（图 1A、图 1B），1例患者为单发性。1例患者伴发左肾转移。

**1:**
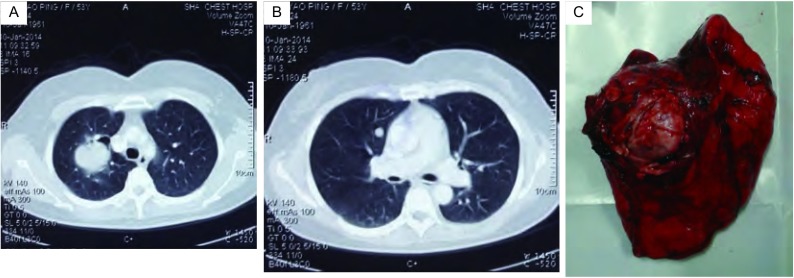
1例患者的临床影像学特征和手术标本。A：胸部CT提示右肺上叶肿块伴偏心性空洞；B：右肺中叶内侧段实性结节；C：手术标本可见肺内实性肿块，包膜完整。 Clinical radiologic features and specimen of one patient. A: Chest computed tomography showing: a tumor with eccentric hole in superior lobe of right lung; B: A solid nodule in medial segment of right middle lobe of lung; C: A mass with complete capsule in superior lobe of right lung of the patient.

手术方式包括4例多发性患者3例行胸腔镜下楔形切除术，1例行右肺上叶切除术+右肺中叶、下叶楔形切除术（图 1C），1例单发性行左肺上叶切除。

术后病理肺内肿瘤细胞均为分化成熟的平滑肌细胞，与原子宫平滑肌瘤细胞形态一致，无细胞异型性、未见坏死和核分裂象。标本雌激素受体（estrogen receptor, ER）、孕激素受体（progesterone receptor, PR）阳性率80%，Ki-67阴性或＜1%。

5例患者随访3个月-48个月，平均22.2个月，均病情稳定，肺内病灶无明显进展，亦未发现肺内新病灶。1例患者术后给予他莫昔芬治疗1年，因雌、孕激素水平高，行手术切除卵巢。

## 2 讨论

肺良性转移性平滑肌瘤临床十分罕见，1939年首先由Steiner报道^[1]^。该病病理起源目前仍存在争议，主要以下几种假说：①多数学者认为肿瘤来源于转移的良性子宫平滑肌瘤^[2]^；②转移自低度恶性的平滑肌肉瘤^[3]^；③多中心生长的平滑肌瘤，认为该瘤应为原发于肺的多发性平滑肌瘤性错构瘤（multiple pulmonary leiomyomatous hamartoma, MPLH）或多发性纤维平滑肌瘤性错构瘤（multiple pulmonary fibroleiomyomatous hamartoma, MPFLH）^[4]^；④肺富含平滑肌的错构瘤。支持肿瘤来源于转移的良性子宫肌瘤的依据有以下几点：①子宫与肺肿瘤均表达雌、孕激素受体；②文献^[5]^报道其对抗雌激素治疗有效；③在遗传学水平上，所有的良性转移性平滑肌瘤患者均出现染色体19q和22q的缺失，部分BML患者伴有1P的缺失。由于该病比较罕见，并且无典型的临床的特征，因此极易被误诊。在此总结本院5例患者的临床病理特征，以期能在胸外科临床诊治中有一定的启示作用。

良性转移性平滑肌瘤好发于女性，文献^[6]^报道发病年龄23岁-77岁，多有子宫肌瘤手术史。肺部出现转移可发生于诊断子宫平滑肌瘤后3个月-20年，平均14.9年^[7]^，也有转移灶与原发灶同时发现的报道^[8]^。大部分患者早期无明显临床症状或仅为咳嗽、胸痛等表现。本组5例患者均为体检时发现病灶，平均发病年龄46.8岁，PBML发生于诊断子宫肌瘤后平均169个月，与文献报道相仿。

影像学检查是诊断肺部转移瘤的主要手段，文献报道PMBL转移瘤主要表现为双肺实性结节。Horstamann等^[2]^回顾性总结了23例PMBL患者的影像学检查发现最常见的为双肺多发性实性结节（16例，70%），少数可有分叶或空洞，其次为单侧多发性肿块（4例，17%），而单侧肿块较少（3例，13%），罕见粟粒样弥漫性病变。北京协和医院冯敏等报道6例患者中有1例患者为囊性肿块^[8]^。本院资料中有1例患者右肺上叶肿块直径约4 cm，伴偏心性空洞。BML转移可发生于全身多处部位，包括皮肤、骨盆、腹部、大网膜、下腔静脉等处，但以肺和淋巴结多见。本组资料中有一例患者双肺多发性结节同时伴有左肾转移，于呼吸内科反复行肺穿刺活检细胞学检查未能明确诊断，后经胸腔镜下活检病理及免疫组化检查诊断明确。因此在临床工作中发现双肺多发性实性肿块甚至伴全身其余部位转移灶或者伴有空洞时，不能盲目诊断为肺癌或非特异性感染病灶，应考虑该病可能，仔细询问病史，尤其是否有子宫肌瘤病史。

该病的最终确诊仍依赖于病理学检查和免疫组化，需与肺原发性平滑肌瘤/肉瘤以及肺转移性平滑肌肉瘤相鉴别。原发性肺平滑肌瘤非常罕见，不表达雌激素受体；而原发性肺平滑肌肉瘤亦罕见，有细胞异型性、坏死，有丝分裂活动活跃，增长迅速，同时这两种肿瘤肺内病灶多为单发。对于转移性平滑肌肉瘤，Kayser等^[7]^报道了2例源于子宫的肺转移性平滑肌肉瘤，在与10例PBML进行对比分析后认为：①PBML常发生于有子宫肌瘤史的绝经前女性(平均年龄45岁)，而子宫平滑肌肉瘤多发生于老年女性；②PBML发生肺转移的时间与手术间隔时间长于肉瘤；③影像学上PBML在肺常表现为多发结节；肉瘤转移至肺的结节多是单发或2个，平均直径大于PBML结节；④PBML雌、孕激素受体的阳性率达80%，而2例转移的平滑肌肉瘤中仅1例表达；⑤p53蛋白质定量分析表明，30%的肉瘤表达p53蛋白，而PBML约为50%；⑥平滑肌肉瘤的Ki-67标记指数（11%）高于PBML（2.9%）。北京协和医院冯敏等^[8]^报道6例PBML患者ER、PR受体阳性率达100%。本组患者中ER、PR阳性率为80%，Ki-67为阴性或＜1%，与文献报道相吻合，肺良性转移性平滑肌瘤诊断明确。

该病临床罕见，目前治疗上仍缺乏统一认识，多数学者认为对于可切除的病灶应首选手术切除，也有助于明确诊断，同时需进行密切随访，观察有无新发病灶。由于该肿瘤表达雌、孕激素受体，为激素依赖型，不少研究认为内分泌治疗应当有效，可行外科卵巢切除或药物性闭经治疗。有文献^[9]^报道给予甲羟孕酮600 ng/d治疗2年后肺部病灶退化；也有文献^[10]^报道1例患者使用米非司酮治疗后，肺内平滑肌成分减少，出现肺泡的纤维化；但也有抗雌激素治疗失败的案例报道。亦有学者^[6]^设想使用伊马替尼治疗，但目前仍缺乏临床证据。本组5例患者均行手术治疗，有2例患者行根治性切除，3例患者胸腔镜下活检，术后1例患者给予了内分泌治疗，说明对于该病目前尚缺乏固定的治疗模式，同时也说明在临床工作中对于该病仍缺乏充分认识。该病多为肺内周围型病变，因此当前胸腔镜手术的广泛开展，为这类患者明确诊断与手术治疗提供了微创手术的可能。

PBML病程缓慢，预后较好。Motegi等^[11]^报道14例PBML患者中1年内死亡的仅有1例，2例生存1年-2年，11例生存期超过4年，最长者生存期超过30年。Kayser等^[7]^报道的10例PBML，病变切除后中位生存期达94个月，而同时观察的2例转移性平滑肌肉瘤最长生存期为22个月，其认为PBML发生的时间间隔长、病灶＜3 cm、肿瘤组织含中等量的血管、激素受体阳性率高的患者预后较好。也有文献^[12]^认为P53表达水平与预后密切相关。

总之，临床医师在工作应对PBML有所重视，工作中对育龄期女性、有子宫肌瘤病史、肺内出现结节或弥漫性病变，需考虑PBML可能。对能根治性切除的病例，应手术切除，不能根治性切除的患者应尽可能对多个病变部位行活检术，明确病理类型及来源，并考虑行内分泌治疗，随访监测患者雌、孕激素水平和肺内病变变化情况。
